# Supplementary material for: MetaQTL: a package of new computational methods for the meta-analysis of QTL mapping experiments
Source: BMC Bioinformatics. 2007 Feb 8;8:49. doi: 10.1186/1471-2105-8-49 (PMC1808479; doi:10.1186/1471-2105-8-49)
Supplement: Additional File 7 — MetaQTL Package : jar file and tutorial. This Zip archive contains both the MetaQTL JAR file and the files of the tutorial. [file 1471-2105-8-49-S7.zip › org.inra.metaqtl/doc/org/metaqtl/QtlPartition.html]

QtlPartition


|  |  |  |
| --- | --- | --- |
| **Overview** \| **Package** \| **Class** \| **Use** \| **Tree** \| **Deprecated** \| **Index** \| **Help** | |  |
| **PREV CLASS**   **NEXT CLASS** | **FRAMES**    **NO FRAMES**     **All Classes** |  |
| SUMMARY: NESTED \| FIELD \| CONSTR \| METHOD | DETAIL: FIELD \| CONSTR \| METHOD |  |


---


## org.metaqtl Class QtlPartition

```
java.lang.Object
  org.metaqtl.QtlPartition
```

---

``` public class QtlPartition extends java.lang.Object ```

---

| **Field Summary** | |
| --- | --- |
| `static int` | `HORIZONTAL_MODE` |
| `int` | `mode`             The mode of representation |
| `int` | `np`             The number of partition. |
| `int` | `nqtl`             The number of Qtl. |
| `java.lang.String[]` | `pnames`             The partition names. |
| `double[][]` | `proba`             The probabilities of each Qtl to belong to each partition. |
| `double[]` | `qtlCI`             The Qtl confidence intervals. |
| `java.lang.String[]` | `qtlNames`             The qtl names |
| `double[]` | `qtlPos`             The Qtl positions |
| `Tree` | `tree`             The Qtl tree. |
| `static int` | `VERTICAL_MODE` |


**Constructor Summary**

`QtlPartition()`


| **Method Summary** | |
| --- | --- |
| `java.util.ArrayList` | `getPartNames()` |
| `void` | `rebuildPartition(java.lang.String[] pnames)` |
| `void` | `setTree(Tree tree)` |

**Methods inherited from class java.lang.Object**

`clone, equals, finalize, getClass, hashCode, notify, notifyAll, toString, wait, wait, wait`

**Field Detail**

### HORIZONTAL\_MODE

```
public static final int HORIZONTAL_MODE
```

**See Also:**: Constant Field Values

---


### VERTICAL\_MODE

```
public static final int VERTICAL_MODE
```

**See Also:**: Constant Field Values

---


### nqtl

```
public int nqtl
```

:   The number of Qtl.

---


### np

```
public int np
```

:   The number of partition.

---


### pnames

```
public java.lang.String[] pnames
```

:   The partition names.

---


### qtlNames

```
public java.lang.String[] qtlNames
```

:   The qtl names

---


### proba

```
public double[][] proba
```

:   The probabilities of each Qtl to
    belong to each partition.

---


### qtlPos

```
public double[] qtlPos
```

:   The Qtl positions

---


### qtlCI

```
public double[] qtlCI
```

:   The Qtl confidence intervals.

---


### tree

```
public Tree tree
```

:   The Qtl tree.

---


### mode

```
public int mode
```

:   The mode of representation


**Constructor Detail**

### QtlPartition

```
public QtlPartition()
```


**Method Detail**

### getPartNames

```
public java.util.ArrayList getPartNames()
```

:   **Returns:**

---


### setTree

```
public void setTree(Tree tree)
```

:   **Parameters:**: `tree` -

---


### rebuildPartition

```
public void rebuildPartition(java.lang.String[] pnames)
```

:   **Parameters:**: `names` -


---


|  |  |  |
| --- | --- | --- |
| **Overview** \| **Package** \| **Class** \| **Use** \| **Tree** \| **Deprecated** \| **Index** \| **Help** | |  |
| **PREV CLASS**   **NEXT CLASS** | **FRAMES**    **NO FRAMES**     **All Classes** |  |
| SUMMARY: NESTED \| FIELD \| CONSTR \| METHOD | DETAIL: FIELD \| CONSTR \| METHOD |  |


---
